# Investigation of *MLH1, MGMT, CDKN2A*, and *RASSF1A* Gene Methylation in Thymomas From Patients With Myasthenia Gravis

**DOI:** 10.3389/fnmol.2020.567676

**Published:** 2020-10-23

**Authors:** Fabio Coppedè, Roberta Ricciardi, Angela Lopomo, Andrea Stoccoro, Anna De Rosa, Melania Guida, Loredana Petrucci, Michelangelo Maestri, Marco Lucchi, Lucia Migliore

**Affiliations:** ^1^Department of Translational Research and of New Surgical and Medical Technologies, Medical Genetics Laboratory, University of Pisa, Pisa, Italy; ^2^Department of Clinical and Experimental Medicine, Neurology Unit, University of Pisa, Pisa, Italy; ^3^Division of Thoracic Surgery, Cardiothoracic and Vascular Surgery Department, Pisa University Hospital, Pisa, Italy; ^4^Department of Surgical, Medical and Molecular Pathology and Critical Care Medicine, University of Pisa, Pisa, Italy

**Keywords:** epigenetics, DNA methylation, CDKN2A, RASSF1A, MLH1, MGMT, thymoma, myasthenia gravis

## Abstract

A feature of thymomas is their frequent association with myasthenia gravis (MG), an autoimmune disease characterized by the production of autoantibodies directed to different targets at the neuromuscular junction. Indeed, almost 30–40% of thymomas are found in patients with a type of MG termed thymoma-associated MG (TAMG). Recent studies suggest that TAMG-associated thymomas could represent a molecularly distinct subtype of thymic epithelial tumors (TETs), but few data are still available concerning the epigenetic modifications occurring in TAMG tissues. The promoter methylation levels of DNA repair (*MLH1* and *MGMT*) and tumor suppressor genes (*CDKN2A* and *RASSF1A*) have been frequently investigated in TETs, but methylation data in TAMG tissues are scarce and controversial. To further address this issue, we investigated *MLH1*, *MGMT*, *CDKN2A*, and *RASSF1A* methylation levels in blood samples and surgically resected thymomas from 69 patients with TAMG and in the adjacent normal thymus available from 44 of them. Promoter methylation levels of *MLH1*, *MGMT*, *CDKN2A*, and *RASSF1A* genes were not increased in cancer with respect to healthy tissues and did not correlate with the histological or pathological features of the tumor or with the MG symptoms. The present study suggests that hypermethylation of these genes is not frequent in TAMG tissues.

## Introduction

Myasthenia gravis (MG) is an autoimmune disease characterized by the production of autoantibodies directed to different targets at the neuromuscular junction, in most of the cases against the nicotinic acetylcholine receptor (AChR+ MG patients; Marx et al., 2015). Thymomas are tumors that originate from epithelial cells of the thymus and are often accompanied by non-neoplastic lymphocytic proliferation. These tumors are classified into five subtypes (A, AB, B1, B2, and B3) according to the tumor cell morphology and to the proportion of associated immature T cells (Marx et al., 2014). The Masaoka–Koga staging system groups thymomas into four stages, namely stage I that includes encapsulated tumors, stages II and III for tumors showing direct local invasion, and stage IV for those with a metastatic spread (Ried et al., 2016). One of the unique features of thymomas is their frequent association with AChR+ MG, so that about 30–40% of thymomas are found in patients with a type of MG termed thymoma-associated MG (TAMG; Marx et al., 2015).

Epigenetic modifications, and particularly the gene silencing resulting from hypermethylation of tumor suppressor and DNA repair genes, play a pivotal role in tumor development and progression (Costa-Pinheiro et al., 2015). A recent whole-genome methylation study has suggested that TAMG-associated thymomas are epigenetically distinct from thymomas that develop in people without MG (Bi et al., 2019). However, the study compared very few samples (Bi et al., 2019), so that larger studies are required to epigenetically characterize those tumors and their different histological subtypes, as well as to understand the links between the epigenetic modifications observed in TAMG tissues and the severity of the MG symptoms.

The methylation levels of the DNA repair and tumor suppressor genes *MLH1*, *MGMT*, *CDKN2A*, and *RASSF1A* have frequently been investigated in thymic epithelial tumors (TETs), including thymomas (Hirabayashi et al., 1997; Suzuki et al., 2005; Chen et al., 2009; Hirose et al., 2009; Mokhtar et al., 2014; Kajiura et al., 2017). Collectively, those studies have shown that hypermethylation of those genes is more frequent in thymic carcinomas than in thymomas, but most of those studies did not clarify if thymoma samples were from patients with TAMG or from patients without MG, while others included very few TAMG-associated thymomas, making it not possible to correlate the observed methylation levels with tumor stage, histology, or MG symptoms; in addition, no data were often available from adjacent normal thymic tissues in order to compare the methylation levels between pathological and healthy samples. Furthermore, most of the available studies have been performed in Chinese or Japanese populations and studies in Caucasians are limited (Hirabayashi et al., 1997; Suzuki et al., 2005; Chen et al., 2009; Hirose et al., 2009; Mokhtar et al., 2014; Kajiura et al., 2017). A recent study was performed on 117 TETs using multiplatform omics analyses and including 97 white, six black, and 12 Asian samples and 32 thymomas from patients with TAMG (Radovich et al., 2018). The study revealed an increased prevalence of aneuploidy among MG+ thymomas, but the MG status was not associated with any methylation signature (Radovich et al., 2018).

The present study was performed to investigate if *MLH1*, *MGMT*, *CDKN2A*, and *RASSF1A* genes are hypermethylated in TAMG-associated thymomas with respect to the normal residual thymuses or circulating lymphocytes of the same patients, and if the methylation patterns of TAMG-associated thymomas exhibit differences in relation to age, gender, and TAMG clinical features.

## Methods

### Study Population

The study was performed on available DNA samples from a previously described dataset composed by 69 patients with AChR+ TAMG of Caucasian origin, collected at the Myasthenia Clinic of the Pisa University Hospital (Lopomo et al., 2016). DNA samples were collected from both blood and surgically resected tumor tissues of the 69 patients and from the adjacent normal tissue, available from 44 of them. Trained neurologists evaluated each patient, and the diagnosis of MG was made based on the clinical symptoms together with positivity to AChR antibodies. All the patients had computed tomography (CT) scans of the chest followed by thymectomy. The medical personnel of the Division of Thoracic Surgery of the Pisa University Hospital assessed thymoma staging and histology. MG symptoms of thymectomy were assessed according to the MG Foundation of America (MGFA) classification (Jaretzki et al., 2000). Ninety-seven percent of the patients were taking steroids at the time of surgical specimen collection. In addition, 10% of them were following immunosuppressant and/or immunomodulatory therapy, 6% followed a cisplatin-based chemotherapy prior to surgery, and 4% underwent radiotherapy. None of the patients included in the study was treated with demethylating agents such as DNA methyltransferase inhibitors (DNMTi). Each patient gave an informed written consent for inclusion in the study that was conducted in accordance with the Declaration of Helsinki and approved by the Ethics Committee of the Pisa University Hospital (Protocol number 21302/2015). Demographic and clinical characteristics of the study population are shown (Table 1).

**Table 1:** Demographic and clinicopathological characteristics of the studied population (adapted from Lopomo et al., 2016).

TAMG patients	Age ± SD (years)	Gender *N* (%)	MG onset *N* (%)	Thymoma histology *N* (%)	Masaoka-Koga stage *N* (%)	MGFA classification *N* (%)	Therapy* *N* (%)
*N* = 69	55.5 ± 13.2	F: 37 (54) M: 32 (46)	<50 years: 26 (38) ≥50 years: 43 (62)	A: 13 (18.8) AB: 13 (18.8) B1: 5 (7.2) B2: 23 (33.4) B3: 8 (11.6) B2–B3: 5 (7.2) NS: 2 (3.0)	I: 7 (10) IIa: 15 (22) IIb: 29 (42) III: 7 (10) IVa: 9 (13) NS: 2 (3)	No symptoms 10 (14) Class I: 18 (26) Class IIa: 4 (6) Class IIb: 12 (17.5) Class IIIb: 17 (24.5) Class IVa: 0 (0) Class IVb: 4 (6) Class V. 0 (0) NS: 4 (6)	Steroids: 67 (97) Immune: 7 (10) Chemo: 4 (6) Radio: 3 (4)

TAMG, thymoma-associated myasthenia gravis; *MG*, myasthenia gravis; *F*, females; *M*, males; *N*, number; *NS*, not specified; *MGFA*, MG Foundation of America, classification at thymectomy; *Immuno*, immunomodulatory/immunosuppressant drugs (azathioprine, methotrexate, intravenous immunoglobulins, cyclosporine); *Chemo*, cisplatin-based chemotherapy; *Radio*, radiotherapy. *Almost all the patients (97%) were taking steroids prior to and/or at the time of surgical specimen collection.

### Assessment of *MLH1*, *MGMT*, *CDKN2A*, and *RASSF1A* Promoter Methylation

**Figure 1:**
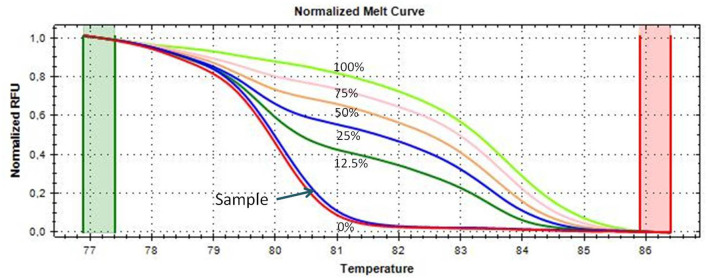
Melting curves of the *CDKN2A* gene generated by samples with known methylation levels (0, 12.5, 25, 50, 75, and 100% methylation, respectively), obtained by mixing the fully methylated and unmethylated standard DNA samples. A thymoma sample showing almost 0% promoter methylation is shown and indicated with an arrow; its melting curve is very similar and partially juxtaposed to that of the completely unmethylated standard DNA (red curve).

An aliquot of 1 ml of peripheral blood in EDTA tubes, obtained by venipuncture, and about 40 mg of tumor tissue and of the adjacent normal tissue, which were clearly distinguishable, obtained during thymectomy, were collected and stored at −20°C until DNA extraction. The adjacent normal tissue was separated from the thymoma by macrodissection and histologically controlled for absence of tumor contamination. Genomic DNA was extracted using the QIAmp DNA Mini Kit (Qiagen, Milan, Italy, Catalog N° 51304) following the manufacturer’s protocol, from 200 μl of whole blood and from about 20 mg of tissue. A Nano Drop ND 2000c spectrophotometer (NanoDrop Thermo Fisher Scientific) was used to quantify the extracted DNA. Two hundred nanograms of DNA from each sample was treated with sodium bisulfite in order to convert all unmethylated cytosines into uracil using the EpiTect Bisulfite Kit (Qiagen, Catalog N° 59104). A sample of completely unmethylated genomic DNA (amplified human genomic DNA, completely unmethylated, Qiagen, Catalog N° 59568) was used as control assay to check the bisulfite conversion efficiency that resulted to be of 99% in average. Promoter methylation was evaluated with methylation sensitive-high resolution melting (MS-HRM) analysis, using primers and protocols previously developed and validated in our laboratory and largely applied by our group for the analysis of gene-specific methylation in human cancers (Migheli et al., 2013; Coppedè et al., 2014). Primer sequences and annealing temperatures (*T*_a_) used during MS-HRM analysis, number of investigated CpG sites, and length of the analyzed amplicons are reported in Table 2. All the methodological details of the MS-HRM analyses can be found elsewhere (Coppedè et al., 2014), and we previously validated the MS-HRM protocols used in this study with pyrosequencing, which is considered the gold standard technique for DNA methylation investigations (Failli et al., 2013; Migheli et al., 2013). Briefly, the MS-HRM analyses were performed using a CFX96 Real-Time PCR detection system (Bio-Rad, Milan) with the following protocol: 1 cycle of 95°C for 12 min, 50 cycles of 95°C for 30 s, gene-specific annealing temperature (Table 2) for 45 s and 72°C for 30 s, followed by an HRM step of 95°C for 10 s and 50°C for 1 min, 65°C for 15 s, and continuous acquisition to 95°C at one acquisition per 0.2°C. PCR was performed in a final volume of 10 μl, containing 5 μl of master mix (Qiagen, Catalog N° 59445), 10 pmol of each primer, and 10 ng of bisulfite-modified DNA template. Each reaction was performed in duplicate, and we analyzed 10% of the samples independently on separate occasions to verify the interassay variability. We mixed fully methylated and unmethylated DNA samples (EpiTect methylated and unmethylated human control DNA, bisulfite converted, Qiagen, Milan, Italy, Catalog N° 59695) to generate standard curves (Figure 1), and the methylation level of each sample was quantified using an interpolation method developed and described in our laboratory (Migheli et al., 2013). Three positive and three negative controls were included in the analyses. Positive controls are DNA samples from patients with colorectal cancer that showed hypermethylation (promoter methylation levels ranging from 70–100%) of the investigated genes in the cancer but not in the adjacent healthy mucosa. Negative controls are DNA samples from patients with colorectal cancer that showed hypomethylation (promoter methylation levels ranging from 1–5%) of the investigated genes in both cancer and adjacent healthy mucosa (Supplementary Figure 1). Both positive and negative controls were selected among DNA samples whose methylation levels have previously been assessed and validated in our laboratory (Coppedè et al., 2014).

**Table 2:** Primer sequences and annealing temperatures (*T*_a_) used during MS-HRM analysis, amplicon length, and number of CpG sites for each gene.

Gene	Primer sequences	*T*_a_	Amplicon length (bp)	CpG sites
*MGMT*	F 5′-GCGTTTCGGATATGTTGGGATAAGT-3′ R 5′-AACGACCCAAACACTCACCAAA-3′	58°	110	12
*MLH1*	F 5′-AGTTTTAAAAACTGAATTAATAGGAAGAG-3′ R 5′-ACTACCCGCTACCTAAAAAAATATAC-3′	56°	81	5
*CDKN2A*	F 5′-CGGAGGAAGAAAGAGGAGGGGT-3′ R 5′-CGCTACCTACTCTCCCCCTCT-3′	62°	93	7
*RASSF1A*	F 5′-TCGGGTTTTATAGTTTTTGTATTTAGGTTTT-3′ R 5′-CCTCCCCCAAAATCCAAACTAA-3′	60°	87	7

### Statistical Analyses

Differences in mean methylation levels among tissues (thymomas, normal thymuses, and blood) were evaluated with analysis of variance (ANOVA). ANOVA was also used to evaluate the effect of clinical, demographic, and pathological features, such as thymoma histology, tumor staging, gender, MG onset, and symptoms, on the mean methylation levels of the studied genes. Linear regression analysis was performed to search for correlation between age and methylation levels. Analyses were performed with the MedCalc statistical software v. 12.5. We used the *post hoc* Bonferroni’s correction tool in MedCalc to correct the *p* values for the multiple comparisons among groups. The statistical power of the study was evaluated with the clinical tool and calculators for medical professionals: ClinCalc^1^. The sample size was chosen to have an *a priori* power of >80% to detect mean methylation differences of 5% or higher among groups. A *post hoc* analysis based on the distribution of methylation data observed in our samples revealed that five samples per group were enough to have 90% power to detect mean DNA methylation differences of 5% or higher among groups, and that including 40 samples per group allowed to have more than 80% power to detect mean DNA methylation differences of 2% or higher among groups.

## Results

**Figure 2:**
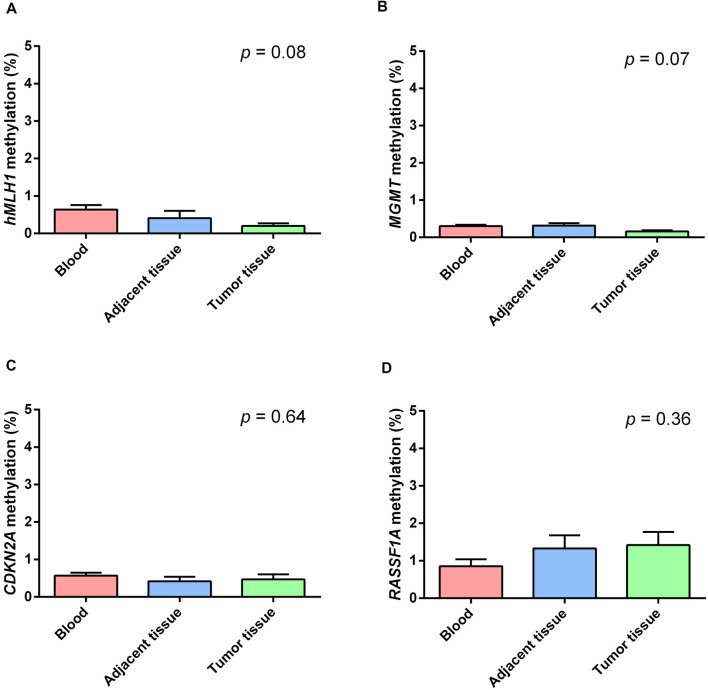
Methylation levels of the studied genes in blood (red), healthy thymic tissue (blue), and tumor tissue (green). **(A)** *MLH1*. **(B)** *MGMT*. **(C)** *CDKN2A*. **(D)** *RASSF1A*. All the investigated genes resulted largely hypomethylated in the studied tissues, and no significant difference among them was observed. Data are shown as means ± SEM (mean standard error), and for each gene, we compared 69 blood samples, 69 thymomas, and 44 healthy thymuses. The *p* value reflects the Bonferroni’s corrected *p* value of the comparison of mean methylation levels among the three groups.

Figure 2 shows the mean methylation levels of the studied regions in blood, tumor tissue, and adjacent healthy thymic tissue of the whole study cohort. MS-HRM analyses showed that *MLH1* methylation levels were very low in peripheral blood (0.64 ± 0.13%), adjacent tumor tissue (0.40 ± 0.19%), and in thymomas (0.19 ± 0.14%), and no overall difference among the three tissues was observed (*p* = 0.08; Figure 2A). Particularly, no differences in *MLH1* mean methylation levels between peripheral blood and adjacent healthy tissue (*p* = 0.68), between peripheral blood and thymomas (*p* = 0.07) and between adjacent tumor tissue and thymomas (*p* = 0.86) were detected. Figure 2B shows the mean methylation levels of *MGMT* in peripheral blood (0.30 ± 0.04%), adjacent healthy tissue (0.32 ± 0.05%), and tumor tissue (0.16 ± 0.05%). Overall, there was no difference among the three tissues (*p* = 0.07), and no differences in *MGMT* mean methylation levels between peripheral blood and adjacent tumor tissue (*p* = 1.00), between peripheral blood and thymomas (*p* = 0.15), and between adjacent tumor tissue and thymomas (*p* = 0.07) were detected. Figure 2C shows the mean methylation levels of *CDKN2A* in peripheral blood (0.57 ± 0.10%), in healthy adjacent tissue (0.42 ± 0.11), and in thymoma tissue (0.47 ± 0.10%). There was no overall difference among the three tissues (*p* = 0.64). No differences in *CDKN2A* mean methylation levels between peripheral blood and adjacent tumor tissue (*p* = 1.00), between peripheral blood and thymomas (*p* = 1.00), and between adjacent tumor tissue and thymomas (*p* = 1.00) were detected. Figure 2D shows the mean methylation levels of *RASSF1A* in peripheral blood (0.84 ± 0.30%), in healthy adjacent tissue (1.32 ± 0.30), and in thymoma tissue (1.41 ± 0.30%). Overall, no significant difference was observed among the three investigated tissues (*p* = 0.36). No differences in *RASSF1A* mean methylation levels between peripheral blood and adjacent tumor tissue (*p* = 0.80), between peripheral blood and thymomas (*p* = 0.56), and between adjacent tumor tissue and thymomas (*p* = 1.00) were detected. In addition, the comparison of the methylation levels observed in the thymoma and in the normal thymus, for each of the 44 patients with available healthy thymus, is shown (Supplementary Figures 2–5). All the investigated genes were largely demethylated in thymomas, and no correlation with the demographic (age and gender) or clinical data (tumor histology and grade) was observed (Supplementary Table 1).

## Discussion

In the present study, we investigated the promoter methylation levels of *MLH1*, *MGMT*, *CDKN2A*, and *RASSF1A* in blood and tumor tissue DNA from 69 patients with TAMG, as well as in the healthy thymic epithelial tissue adjacent to the tumor available from 44 of them. The promoters of *MLH1*, *MGMT*, *CDKN2A*, and *RASSF1A* genes resulted completely demethylated in the studied samples, and no difference in average promoter methylation levels was observed in the comparison of cancerous and healthy thymic tissue. Similarly, no correlation with demographic data of the patients or clinical characteristics of the tumors was found.

We decided to investigate these four genes because their methylation levels have frequently been investigated in TETs, but their methylation levels in TAMG-associated thymomas are still scarce. Collectively, previous studies in TETs have shown that these genes are frequently hypermethylated in thymic carcinomas, but studies investigating their methylation levels in thymomas have provided conflicting and inconclusive results. Moreover, those studies included very few TAMG-associated thymomas (Hirabayashi et al., 1997; Suzuki et al., 2005; Chen et al., 2009; Mokhtar et al., 2014; Kajiura et al., 2017). Also genome-wide methylation data in TAMG are conflicting. Indeed, the comparison of two TAMG-associated thymomas and four thymomas from people without MG revealed that the DNA methylation profiling of MG-associated thymomas was significantly distinguished from that of non-MG thymomas (Bi et al., 2019). However, a larger study on 117 TET samples, that included 32 thymomas from patients with TAMG, showed that the MG status was not associated with any methylation signature (Radovich et al., 2018). These genome-wide studies, however, do not show details on the methylation levels of the four genes investigated in the present study, namely *MGMT*, *MLH1*, *CDKN2A*, and *RASSF1A*.

For all these reasons, we designed the present study to deeply investigate *MGMT*, *MLH1*, *CDKN2A*, and *RASSF1A* methylation levels in a cohort of Caucasian TAMG samples large enough to evaluate the contribution of tumor stage and histology, as well as those of the MG clinical subtypes, to the observed findings. Our investigation revealed that all of *MLH1*, *MGMT*, *CDKN2A*, and *RASSF1A* gene promoters were completely demethylated in TAMG samples, with average methylation levels of about 1–2%. At best of our knowledge, no previous study compared *MLH1*, *MGMT*, *CDKN2A*, and *RASSF1A* gene methylation levels among thymoma samples, healthy thymic samples, and circulating blood DNA from the same patients, and the present study revealed that *MLH1*, *MGMT*, *CDKN2A*, and *RASSF1A* genes showed similar and very low methylation patterns in the three investigated tissues, and no difference was observed between pathological and healthy thymus. Similarly, no correlation was found between gene methylation levels and the histological or clinical characteristics of the tumors. Unfortunately, we could not collect RNA samples from our patients in order to perform gene expression analysis. However, we used quantitative MS-HRM protocols previously validated with pyrosequencing (Migheli et al., 2013; Coppedè et al., 2014) and/or with DNA methylation arrays (Coppedè et al., 2017). These protocols were able to quantify from very low methylation levels of about 0–1% up to 80–100% promoter methylation (Coppedè et al., 2014, 2017), and we previously demonstrated that very low methylation levels of these genes (1–2% in average) are found in healthy tissues, including blood DNA from healthy people or the healthy colonic mucosa (Coppedè et al., 2014, 2017). By contrast, methylation levels of 80% or higher were frequently observed in colorectal cancer specimens, resulting in loss of protein expression (Coppedè et al., 2014). Collectively, the present study suggests that the promoter methylation levels of *MLH1*, *MGMT*, *CDKN2A*, and *RASSF1A* genes are very low in TAMG samples and are comparable with those observed in healthy tissues. A previous study performed by us in the same cohort revealed no difference in the methylation levels of DNA methyltransferase genes (*DNMT1*, *DNMT3A*, and *DNMT3B*) between thymomas and healthy thymus (Lopomo et al., 2016). A more recent investigation in the same cohort revealed that the *GHSR* gene was modestly hypermethylated only in less than 30% of the samples (Coppedè et al., 2020), while it resulted strongly hypermethylated in thymic carcinomas (Kishibuchi et al., 2020).

One of the limits of the present study is the unavailability of thymoma samples from individuals without MG to make comparisons, so that we cannot understand whether the almost-complete absence of methylation of the investigated genes is a unique feature of TAMG-associated thymomas or not. Larger studies are therefore needed, investigating all in one point mutations, chromosome rearrangements, and epigenetic signatures of the different TET subtypes. Another limitation of the study is that all but two patients were taking corticosteroids at the time of sample collection or before. The methylation levels observed in these two patients are not dissimilar from those observed in individuals taking corticosteroids (Supplementary Figures 2–5), and all the investigated genes were demethylated in their tissues. Further studies are however required to clarify if corticosteroid treatment could impair the methylation levels of the studied genes. Previous studies in Asian samples have shown that *MLH1*, *MGMT*, *CDKN2A*, and *RASSF1A* genes are hypomethylated in thymomas as compared with thymic carcinomas or neuroendocrine tumors (Hirabayashi et al., 1997; Suzuki et al., 2005; Chen et al., 2009; Mokhtar et al., 2014; Kajiura et al., 2017). We have not included thymic carcinomas or neuroendocrine tumors in the present investigation to make comparisons, but all the four genes were scarcely methylated in the thymoma samples from Caucasian patients with TAMG included in the present study.

In summary, the present study reveals that the promoter methylation levels of *MLH1*, *MGMT*, *CDKN2A*, and *RASSF1A* genes are not increased in thymomas with respect to the healthy tissues of patients with MG and do not correlate with histological or pathological features.

## Data Availability Statement

The raw data supporting the conclusions of this article will be made available by the authors, without undue reservation.

## Ethics Statement

The studies involving human participants were reviewed and approved by Pisa University Hospital Ethics Committee. The patients/participants provided their written informed consent to participate in this study.

## Author Contributions

FC conceived and designed the study, provided reagents and funds, and wrote the article. LM critically reviewed the manuscript. AL performed methylation experiments. AS supervised methylation experiments and performed the statistical analysis. RR, AD, LP, MG, and MM performed neurological examinations of the patients and disease diagnosis. ML evaluated thymic pathology. All authors contributed to the article and approved the submitted version.

## Conflict of Interest

The authors declare that the research was conducted in the absence of any commercial or financial relationships that could be construed as a potential conflict of interest.

## Footnotes

^1^http://clincalc.com/Stats/Power.aspx
