# Supplementary material for: Investigation of MLH1, MGMT, CDKN2A, and RASSF1A Gene Methylation in Thymomas From Patients With Myasthenia Gravis
Source: Front Mol Neurosci. 2020 Oct 23;13:567676. doi: 10.3389/fnmol.2020.567676 (PMC7645111; doi:10.3389/fnmol.2020.567676)
Supplement: Supplementary file 1 [file Data_Sheet_1.docx]

**Supplementary Table.** Correlation of methylation levels with demographic and clinical data

| **Gene** | **Age** | **Gender** | **Histology** | **Masaoka-Koga classification** |
| --- | --- | --- | --- | --- |
| *MGMT* | *P*=0.61  r=0.06 | *P*=0.89 | *P*=0.91 | *P*=0.39 |
| *MLH1* | *P*=0.37  r=0.11 | *P*=0.34 | *P*=0.33 | *P*=0.74 |
| *CDKN2A* | *P*=0.47  r=0.08 | *P*=0.29 | *P*=0.36 | *P*=0.47 |
| *RASSF1A* | *P*=0.34  r=0.11 | *P*=0.78 | *P*=0.35 | *P*=0.62 |

**Supplementary Figure 1**

**
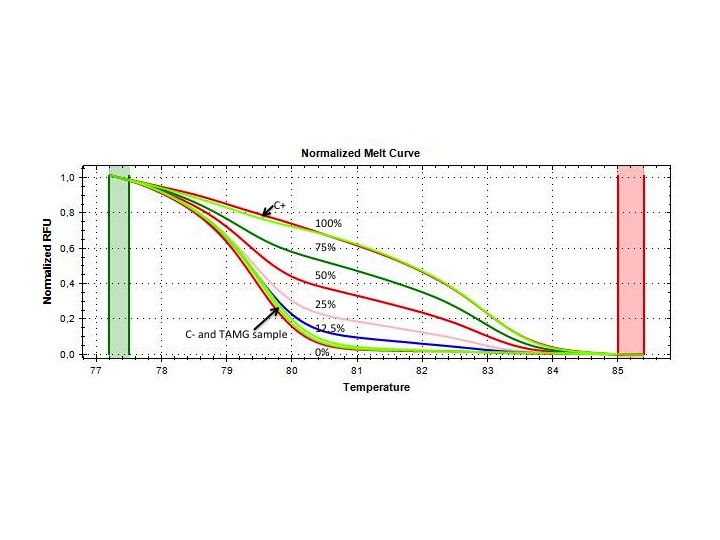
**

**Figure Legend:**

Fig. 1. Melting curves of the *CDKN2A* gene generated by samples with known methylation levels (0%, 12.5%, 25%, 50%, 75%, and 100% methylation, respectively), obtained by mixing the fully methylated and unmethylated standard DNA samples. A TAMG+ thymoma sample (TAMG sample), a methylated control (C+) and a demethylated control (C-) are shown. The thymoma sample and the C- are largely demethylated and their melting curves (indicated with the arrow) are very similar to that of the completely unmethylated standard DNA (0% methylation). The melting curve of the positive control (C+ indicated with the arrow) is juxtaposed to that of the completely methylated standard (100% methylation).

**Supplementary Figure 2**

**Figure legend:** Methylation levels of the *MLH1* gene in the 44 TAMG-associated thymoma samples with available healthy thymus. Each sample is indicated with a different number, and the methylation levels are indicated in blue (healthy thymus) or orange (thymoma). The methylation levels of the three positive (C+) and negative (C-) controls are shown for comparison (in blue the healthy tissue and in orange the tumor tissue). In this cohort of matched thymomas and healthy thymuses from the same patients, the mean *MLH1* methylation levels (%) were 0.30 ± 0.10 in thymomas vs. 0.40 ± 0.19 in healthy thymuses (*p* = 0.59). Samples 8 and 25 are from individuals not taking corticosteroids.

**Supplementary Figure 3**

**Figure legend:** Methylation levels of the *MGMT* gene in the 44 TAMG-associated thymoma samples with available healthy thymus. Each sample is indicated with a different number, and the methylation levels are indicated in blue (healthy thymus) or orange (thymoma). The methylation levels of the three positive (C+) and negative (C-) controls are shown for comparison (in blue the healthy tissue and in orange the tumor tissue). In this cohort of matched thymomas and healthy thymuses from the same patients, the mean *MGMT* methylation levels (%) were 0.22 ± 0.06 in thymomas vs. 0.32 ± 0.05 in healthy thymuses (*p* = 0.08). Samples 8 and 25 are from individuals not taking corticosteroids.

**Supplementary Figure 4**

**Figure legend:** Methylation levels of the *CDKN2A* gene in the 44 TAMG-associated thymoma samples with available healthy thymus. Each sample is indicated with a different number, and the methylation levels are indicated in blue (healthy thymus) or orange (thymoma). The methylation levels of the three positive (C+) and negative (C-) controls are shown for comparison (in blue the healthy tissue and in orange the tumor tissue). In this cohort of matched thymomas and healthy thymuses from the same patients, the mean *CDKN2A* methylation levels (%) were 0.47 ± 0.13 in thymomas vs. 0.42 ± 0.11 in healthy thymuses (*p* = 0.78). Samples 8 and 25 are from individuals not taking corticosteroids.

**Supplementary Figure 5**

**Figure legend:** Methylation levels of the *RASSF1A* gene in the 44 TAMG-associated thymoma samples with available healthy thymus. Each sample is indicated with a different number, and the methylation levels are indicated in blue (healthy thymus) or orange (thymoma). The methylation levels of the three positive (C+) and negative (C-) controls are shown for comparison (in blue the healthy tissue and in orange the tumor tissue). In this cohort of matched thymomas and healthy thymuses from the same patients, the mean *RASSF1A* methylation levels (%) were 1.42 ± 0.35 in thymomas vs. 1.32 ± 0.30 in healthy thymuses (*p* = 0.85). Samples 8 and 25 are from individuals not taking corticosteroids.
